# Use of dynamic contrast-enhanced MRI to evaluate acute treatment with ZD6474, a VEGF signalling inhibitor, in PC-3 prostate tumours

**DOI:** 10.1038/sj.bjc.6601386

**Published:** 2003-11-11

**Authors:** D Checkley, J J Tessier, J Kendrew, J C Waterton, S R Wedge

**Affiliations:** 1 Department of Enabling Science and Technology, AstraZeneca, Mereside, Alderley Park, Macclesfield, Cheshire SK10 4TG, UK; 2 Department of Cancer & Infection Research, AstraZeneca, Mereside, Alderley Park, Macclesfield, Cheshire SK10 4TG, UK

**Keywords:** VEGF, tyrosine kinase inhibitor, ZD6474, vascular permeability, DCE-MRI, gadopentetate dimeglumine

## Abstract

Dynamic contrast-enhanced magnetic resonance imaging (DCE-MRI), using gadopentetate dimeglumine, was used to monitor acute effects on tumour vascular permeability following inhibition of vascular endothelial growth factor-A (VEGF-A) signal transduction. Mice bearing PC-3 human prostate adenocarcinoma xenografts were treated with ZD6474, a VEGF receptor-2 (KDR) tyrosine kinase inhibitor. The pharmacokinetic parameter *K*^trans^ was obtained, which reflects vascular permeability and perfusion. Mice were imaged immediately before, and following, acute treatment with ZD6474 (12.5–100 mg kg^−1^ orally). Whole tumours were analysed to obtain mean *K*^trans^ values, and a histogram approach was used to examine intratumour heterogeneity. Reproducibility of *K*^trans^ measurements gave inter- and intra-animal coefficients of variation of 40 and 18%, respectively. Dose-related reductions in *K*^trans^ were evident following acute ZD6474 treatment. A *K*^trans^ reduction of approximately 30% (*P*<0.001) was evident with 50 and 100 mg kg^−1^ ZD6474, a reduction of 12.5% (*P*<0.05) at 25 mg kg^−1^, and a reduction that did not reach statistical significance at 12.5 mg kg^−1^. A correlation between this dose response and the growth inhibitory effect of ZD6474 following chronic treatment was also observed. The histogram analysis of the data indicated that ZD6474-induced a *K*^trans^ reduction in both the most enhancing rim and the core of PC-3 tumours. Dynamic contrast-enhanced magnetic resonance imaging may have a role in assessing the acute effects of VEGF signalling inhibition, in clinical dose-ranging studies.

Vascular endothelial growth factor-A (VEGF) has a key role in inducing the pathological angiogenesis required to sustain solid tumour growth. Vascular endothelial growth factor-A expression is elevated in tumours in response to a variety of stimuli (Rak *et al*, 1995; Dibbens *et al*, 1999; Igarashi *et al*, 2002), and its activity is largely restricted to vascular endothelial cells via two high-affinity receptors, Flt-1 (VEGF-R1) and KDR/Flk-1 (VEGF-R2). There are data to suggest that activation of KDR alone is sufficient to promote endothelial cell migration, proliferation and tubule formation (Meyer *et al*, 1999; Gille *et al*, 2001; Zeng *et al*, 2001).

In addition to its proangiogenic effects on endothelial cells, VEGF has a profound permeabilising effect on the vasculature and increases vasodilation (Bates *et al*, 2002; Dafni *et al*, 2002), which may also contribute to disease progression by enhancing tumour nutrient and waste exchange. In keeping with other VEGF-induced phenotypes, VEGF-dependent vascular permeability is also thought to be contingent on activation of KDR. This dependency is suggested by studies that have employed ligands with differential selectivity for KDR *vs* Flt-1 (Joukov *et al*, 1998; Ogawa *et al*, 1998; Gille *et al*, 2001; Hillman *et al*, 2001) or KDR-blocking antibodies (Wu *et al*, 1999; Brekken *et al*, 2000). Recently, placental growth factor (PlGF), a selective Flt-1 ligand, has been shown to enhance the vascular permeabilising effect of VEGF (Carmeliet *et al*, 2001; Luttun *et al*, 2002). However, it is conceivable that this phenomenon may involve a cooperative effect with KDR, either via cross receptor signalling, or by PlGF displacement of VEGF already bound to Flt-1, thereby increasing the VEGF that is accessible for binding to KDR.

A number of mechanism-based approaches to inhibit VEGF signal transduction are currently in clinical development. Each of these strategies is likely to require protracted dose administration to prevent angiogenesis and suppress tumour growth. Since disease stabilisation, or increased time to progression, is an anticipated therapeutic outcome, biological markers of activity are being sought to provide dose–response information earlier in the course of treatment. In this regard, imaging methodologies are an attractive prospect, given their noninvasive nature and the potential to provide functional information (Libutti *et al*, 1999). In particular, dynamic contrast-enhanced magnetic resonance imaging (DCE-MRI), where the uptake and washout of a contrast agent in tissues is monitored with time, can provide physiological information on vascular permeability, volume of interstitial space and perfusion. Computed tomography (CT) has also been used in this way (Miles, 2002), although, in contrast to DCE-MRI, the frequency with which CT can be used is restricted by the accumulative dose of radiation that can be administered safely. While DCE-MRI may not be applicable to all antiangiogenic approaches, it could be amenable to monitoring VEGF signalling inhibition, given that VEGF can render tumour vessels hyperpermeable.

ZD6474 is a novel, small molecular weight inhibitor of KDR tyrosine kinase activity and VEGF-stimulated endothelial cell proliferation *in vitro* (Hennequin *et al*, 2002). The compound is active orally, is well tolerated following chronic administration and produces significant inhibition of tumour growth in a range of models *in vivo* (Wedge *et al*, 2002). Clinical evaluation of ZD6474 is currently ongoing in patients with a range of solid tumours (Hurwitz *et al*, 2002). This study examined the ability of DCE-MRI to measure permeability changes, following acute administration of ZD6474 in a human PC-3 prostate tumour model. In order to produce MRI measurements that were independent of machine settings, data were analysed using a pharmacokinetic model, with the transfer constant (*K*^trans^) being measured as an indicator of vascular permeability (Tofts *et al*, 1999). Although the majority of preclinical DCE-MRI studies examining VEGF-signalling inhibition have previously utilised albumin-gadopentetate dimeglumine (Gd-DTPA) (Pham *et al*, 1998; Brasch *et al*, 2000; Gossmann *et al*, 2000), there are no large molecular weight contrast agents currently licensed for use in man. Low molecular weight gadopentetate dimeglumine (Gd-DTPA) was therefore employed because of its wide availability and established clinical use. In contrast to other studies, a voxelwise multislice tumour analysis was performed, rather than using small region of interest (ROI) or single slice data. In addition, to avoid sampling errors and enable exploration of intratumour heterogeneity, both whole tumour mean values and histogram analyses were examined.

The data indicate that significant reductions in *K*^trans^ can be measured in PC-3 tumours following acute ZD6474 treatment. Furthermore, the magnitude of the change in *K*^trans^ is dose-related (12.5–50 mg kg^−1^ ZD6474) and can be correlated with the effect on PC-3 tumour growth induced by more chronic ZD6474 treatment. This study also highlighted the utility of using a histogram method to analyse DCE-MRI data, with greater effects on *K*^trans^ being evident in the core of the tumour.

## MATERIALS AND METHODS

### Xenograft model and antitumour studies

All *in vivo* studies were performed in full compliance with the UK Animals (Scientific Procedures) 1986 Act. PC-3 human prostate adenocarcinoma xenografts were established in athymic mice as described previously (Wedge *et al*, 2000). Briefly, female Swiss athymic mice, at least 8-weeks old, were injected subcutaneously with 10^6^ cells into the hind flank. Tumour volumes were estimated from twice-weekly bilateral Vernier caliper measurements using the formula (length × width) × √ (length × width) × (*π*/6), taking length to be the longest diameter across the tumour and width the corresponding perpendicular. We have previously shown that there is a good correlation between tumour volumes determined by caliper and MRI measurement (Waterton *et al*, 1997), However, an assessment of tumour volume by MRI was not employed in this study because slice thickness (2 mm) was large relative to the tumour dimensions, and use of caliper measurements enabled a direct comparison with volume estimates in chronic tumour growth studies. Tumours reached a volume (mean±s.e.) of 1.22±0.08 cm^3^ in MRI studies and 0.98±0.04 cm^3^ in chronic tumour growth studies, before mice were randomised into groups and treated with ZD6474 or vehicle. ZD6474 was suspended in a 1% (v v^−1^) solution of polyoxyethylene sorbitan monoleate in deionised water and the animals were dosed once daily by oral gavage at 0.1 ml 10^−1^ g body weight. In tumour growth studies, ZD6474 or vehicle were administered once daily for 11 days.

### MRI studies

The Tofts and Kermode model considers contrast moving passively between two compartments; plasma and the extracellular space (*V*_E_). The transfer constant for this movement (*K*^trans^) is dependent on the blood flow, the surface area and the permeability of the vasculature. The concentration of Gd-DTPA in plasma and tissue is determined from the change in *T*_1_ relaxation and the relaxivity of Gd-DTPA in plasma and tissue. The input function measured in the abdominal aorta is assumed to be equivalent to that in the tumour vasculature.

Three separate experiments were carried out that included control groups given vehicle alone, and one or more treatment groups given ZD6474 at 12.5, 25, 50 or 100 mg kg^−1^ day^−1^. Dynamic contrast-enhanced magnetic resonance imaging was performed twice, before and after treatment with ZD6474, with the second DCE-MRI under terminal anesthesia. The treatment regimen was two doses, given 24 and 2 h prior to the second DCE-MRI. Animals were anesthetised with 1–1.5% halothane (‘Fluothane’, AstraZeneca, Macclesfield, UK) for approximately 2 h during which they were mounted in a lidded plastic cradle fitted with a thermocouple and electrocardiogram electrodes. Under a flow of warm air, a heparinised 26-gauge catheter was inserted into the tail vein and attached to a syringe containing 30 mM Gd-DTPA (‘Magnevist’, Schering, Berlin, Germany) in sterile water. The mice were then transferred, in the cradle, to the bore of a horizontal magnet, where their temperature was maintained at 38.0±0.5°C by a flow of warm air, and their heart rates were monitored.

### MRI acquisition protocol

Dynamic contrast-enhanced magnetic resonance imaging was carried out on an instrument incorporating a 63 mm bore quadrature birdcage coil and a 4.7 Tesla horizontal-bore magnet interfaced to a spectrometer (^UNITY^*Inova*, Varian Medical Systems, Palo Alto, CA, USA). The precontrast acquisition protocol comprised a heavily *T*_*2*_-weighted multislice fast spin-echo sequence with a repetition time (TR) of 3000 ms and an effective echo time (TE) of 120 ms. Four slices were obtained covering the whole tumour volume with a resolution of 0.625 × 0.312 × 2 mm (this resolution was employed throughout with a matrix of 512 × 256 × 4). This was followed by a saturation-recovery sequence for *T*_*1*_ mapping of the tumour that consisted of a series of 4 multislice spin-echo image sets (TR=120, 500, 2000 and 10 000 ms; TE=10 ms). Repeated *T*_1_-weighted spin-echo imaging (TR=120 ms, TE=10 ms) was then used to generate multislice dynamic data. Spin-echo images were employed as opposed to the faster gradient-echo, as spin-echo approaches are less prone to confounds from radiofrequency inhomogeneity in assessment of *T1* changes than are gradient echoes. Spin -echoes were also found to give a better signal-to-noise ratio and therefore increased the accuracy of voxel by voxel data fitting. Following acquisition of five dynamic data sets, Gd-DTPA solution (0.01 ml g^−1^ body weight) was manually injected over 3 s into the tail vein of the animals. The dynamic run then continued with repetitions every 16 s for 11 min after the injection, providing 40 image sets. A calibration curve was prepared by measuring the longitudinal relaxation time (*T*_1_) of filtered mouse blood plasma solutions containing different Gd-DTPA concentrations. The concentrations ranged from 0.0–1.8 mM and were measured at 38°C using a saturation-recovery spin-echo imaging sequence. The calibration curve was used to obtain the relaxivity of Gd-DTPA. A vascular input function, obtained previously in the same animal model, and modelled as a biexponential decay, was employed:


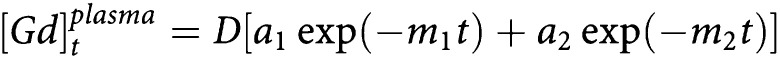


where *D*=0.3 mmol kg^−1^, the bolus dose of Gd-DTPA; the terms *a*_1_=11.95 kg l^−1^ and *m*_1_=0.0195 represent the equilibration of Gd-DTPA between plasma and extracellular space; *m*_2_=0.0009 reflects the renal clearance of Gd-DTPA; and (*a*_1_+*a*_2_)^−1^=16.62 kg l^−1^ represents the plasma volume of the mouse.

### Image processing and analysis

Image processing and analysis was performed with programs written in-house in C and IDL (Research Systems Inc, Boulder, CO, USA), running on a unix workstation. Region of interest comprised entire tumour volumes and were defined using the *T*_*2*_-weighted images to include tumour but exclude surrounding skin. Time courses for contrast enhancement for each voxel within the whole tumour were used to produce parametric maps for analysis using the pharmacokinetic model of Tofts and Kermode (1991). This allowed voxelwise estimations of the volume transfer constant for Gd-DTPA between blood plasma and the extravascular extracellular space (*K*^trans^), and of the volume of extravascular extracellular space per unit volume of tissue (*V*_E_) (Tofts *et al*, 1999). On the basis of their low Gd-DTPA uptake, unfitted voxels were included in the *K*^trans^ maps as zeros.

### Statistical analysis of MRI data

Tumour data were analysed both by grouping all values within treatment groups (*t*-test) and by comparing mean individual animal data obtained pre- and post-treatment (paired *t*-test). The reproducibility of each pairwise comparison in control animals was assessed from the test-retest coefficient of variation (CV). For each subject, *i*, the CV is the standard deviation, *σ*_*i*_, for the two measurements on that subject, divided by the mean volume, *μ*_*i*_, for the subject. The overall test-retest CV for a group of *N* subjects is then:


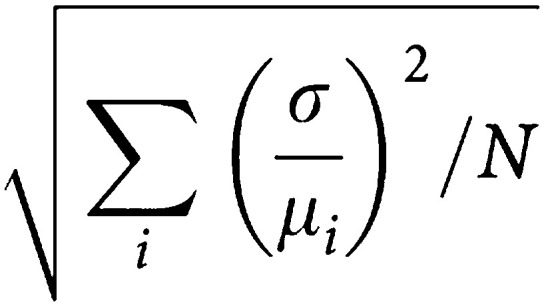


To address the heterogeneous enhancement in tumours (i.e. a rapidly enhancing rim and slowly enhancing core), a novel histogram-based analysis was made by pooling all the *K*^trans^ values from pharmacokinetic model-fitted voxels obtained before and after treatment for individual mice. A threshold of the top 20% of values for *K*^trans^ was chosen that corresponded visually with tumour rims (top 20%) and cores (bottom 80%). Changes in the number of fitted voxels over the 20% threshold pre- and post-treatment with ZD6474 were calculated on a mouse-by-mouse basis and examined using a paired *t*-test. This enabled the effect of drug treatment on the most rapidly enhancing rim (high *K*^trans^ values) and the slower enhancing core (low *K*^trans^ values) to be examined. The exercise was repeated to examine the ZD6474-induced changes in the number of voxels below the 20% threshold. A significance level of 0.05 was used throughout.

## RESULTS

### Qualitative assessment of DCE-MRI images

**Figure 1:**
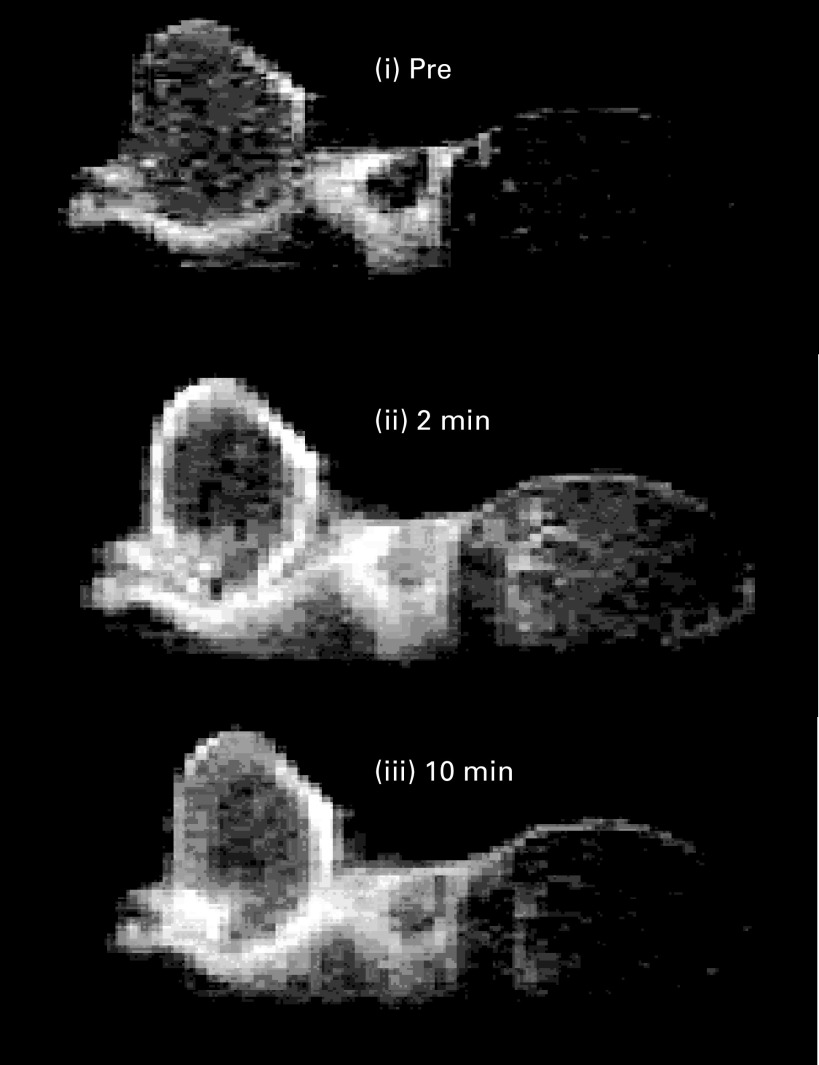
Sagittal single slice images through the tumour and muscle of the hind limb. Dynamic contrast-enhanced magnetic resonance imaging images were acquired before, and 2 and 10 min after, injection of the contrast agent (Gd-DTPA). The tumour rim enhanced rapidly and the core more slowly. Muscle of the hind limb is located to the right of the tumour.

An example of a baseline tumour image (single slice), preceding ZD6474 treatment, is shown in Figure 1

before, and 2 and 10 min after, Gd-DTPA injection. Fast contrast enhancement was observed in peripheral regions of the tumour. Signal enhancement was slower in central regions of the tumour, still increasing in some areas by 11 min after Gd-DTPA injection when the collection of dynamic data was stopped. The effect in the tumour core is suggestive of an enhancement pattern dominated by diffusion. In contrast to tumour, muscle of the hind limb was observed to enhance more uniformly and with loss of signal by the end of the dynamic run: an observation that is consistent with this tissue being comparatively well perfused.

### DCE-MRI data fitting and reproducibility

**Figure 2:**
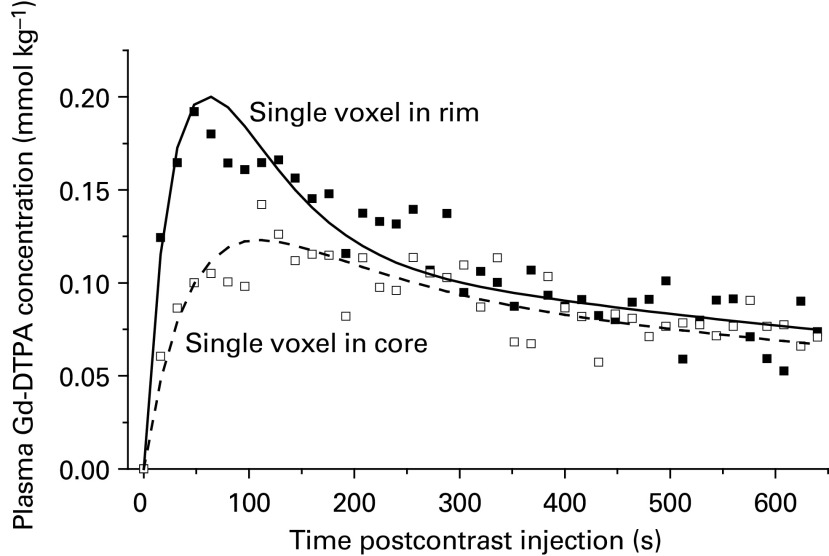
Plot of the concentration of contrast agent (mmol kg^−1^) in mouse plasma against time in seconds after the contrast injection, for a single voxel in the rim and a voxel in the core of the tumour. Curves fitted to the data were generated using the Tofts and Kermode model. The respective rim and core *K*^trans^ values were 1.9 and 0.71 × 10^−3^ s^−1^, while *V*_E_ values were 0.0660 and 0.0661.

A sample kinetic plot of contrast agent concentration for single voxels (selected at random) in the rim and the core of a tumour are shown in Figure 2

. Curves fitted to the data were generated using the Tofts and Kermode model. A clear difference in the rate of initial enhancement was observed, which is consistent with the qualitative assessment of the DCE-MRI images (i.e. greatest enhancement in voxels within the tumour rim).

To estimate the reproducibility of the DCE-MRI method, repeat measurements of 11 vehicle-only-treated control animals were used. Whole tumours were defined from *T*_2_-weighted fast spin- echo images and time courses for enhancement through the tumour enabled voxelwise calculation of pharmacokinetic parameters. There were no significant differences in repeat measurements of mean *K*^trans^ (*P*=0.40) or *V*_E_ (*P*=0.10) made on the same tumour. There was some variability between animals, with inter animal CVs of 40 and 18% for *K*^trans^ and *V*_E_, respectively (Table 1

**Table 1:** Reproducibility of dynamic MRI data

**Parameter**	**Variability^a^**	**Mean±s.d.^b^**	**CV (%)**
*K*^trans^	Between animals	0.54±0.220	40
	Within animals	0.54±0.080	16
*V*_E_	Between animals	0.12±0.021	18
	Within animals	0.12±0.008	7

a Two repeat measurements were made in each of 11 tumour-bearing animals.

b Unit for *K*^trans^ is × 10^−3^ s^−1^.

). This was larger than the intra-animal (i.e. test, retest) variability observed, which gave CVs of 18% for *K*^trans^ and 7% for *V*_E_. Since using each animal as its own control reduced variability by more than 50%, pharmacokinetic data were analysed in this way and group sizes were significantly reduced.

### Acute effects of ZD6474 on tumour enhancement

**Figure 3:**
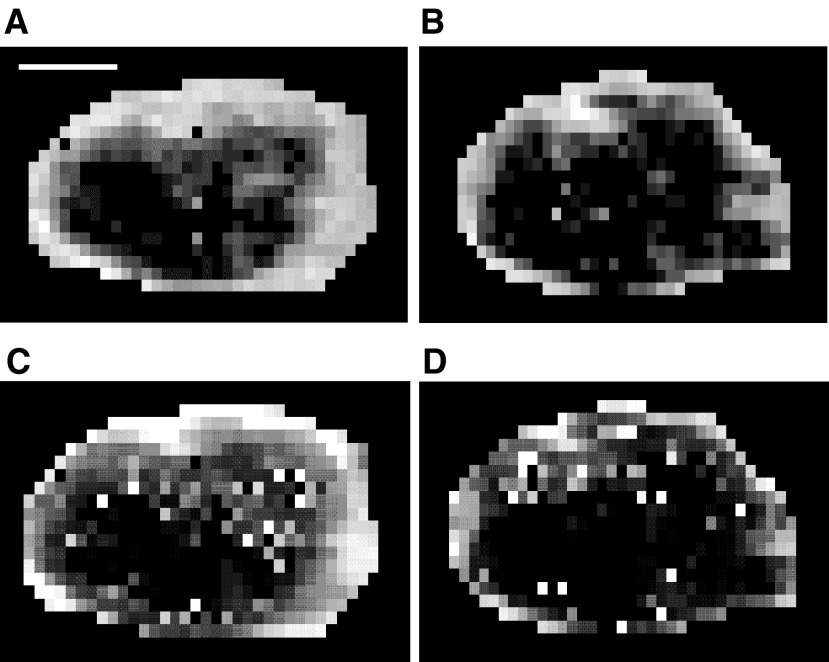
*K*^trans^ (**A**,**B**) and *V*_E_ (**C**,**D**) maps for matched slices through the centre of one tumour obtained pre (**A**,**C**) and post (**B**,**D**) dosing with 50 mg kg^−1^ of ZD6474. White areas have high values for *K*^trans^ or *V*_E_. Grey voxels have low values and black voxels are those that could not be fitted to the Tofts and Kermode model generally as a result of very slow washin and washout of contrast during the study period. The white bar in (**A**) is 3 mm long.

During the 2 days of the MRI experiments, there were no effects of ZD6474 on tumour volumes and no significant change in the percentage of poorly enhancing areas (i.e. unfitted voxels) that may reflect the extent of tumor necrosis. The *K*^trans^ and *V*_E_ maps for matched slices through the centre of a tumour obtained pre- and postdosing with 50 mg kg^−1^ of ZD6474 are shown in Figure 3

: reductions in both parameters were evident following compound treatment. Pooling all pharmacokinetic data from tumours within a treatment group showed that acute ZD6474 treatment reduced mean *K*^trans^ and *V*_E_ at all doses examined, with statistical significance at ⩾25 mg kg^−1^ for *K*^trans^ and ⩾50 mg kg^−1^ for *V*_E_ (Table 2

**Table 2:** Acute effects of ZD6474: changes in *K*^trans^ and *V*_E_ values pre- and postdosing prostate tumour-bearing mice with ZD6474

		**Mean *K*^trans^ (× 10^−3^ s^−1^)^a^**			**Mean *V*_E_a**		
**Dose (mg^−1^/kg)**	**No mice**	**Pre**	**Post**	***P***	**Pre**	**Post**	***P***
0	11	0.54±0.22	0.52±0.21	0.4	0.12±0.02	0.11±0.02	0.1
12.5	6	0.69±0.10	0.58±0.19	0.1	0.11±0.01	0.10±0.02	0.09
25	6	0.56±0.16	0.49±0.17	0.04	0.12±0.02	0.10±0.01	0.07
50	10	0.72±0.19	0.51±0.15	0.0005	0.12±0.01	0.10±0.02	0.0001
100	11	0.62±0.18	0.43±0.10	0.0004	0.14±0.01	0.11±0.01	<0.0001

a Values are grouped means with s.d. from all the animals studied.

; Figure 4

**Figure 4:**
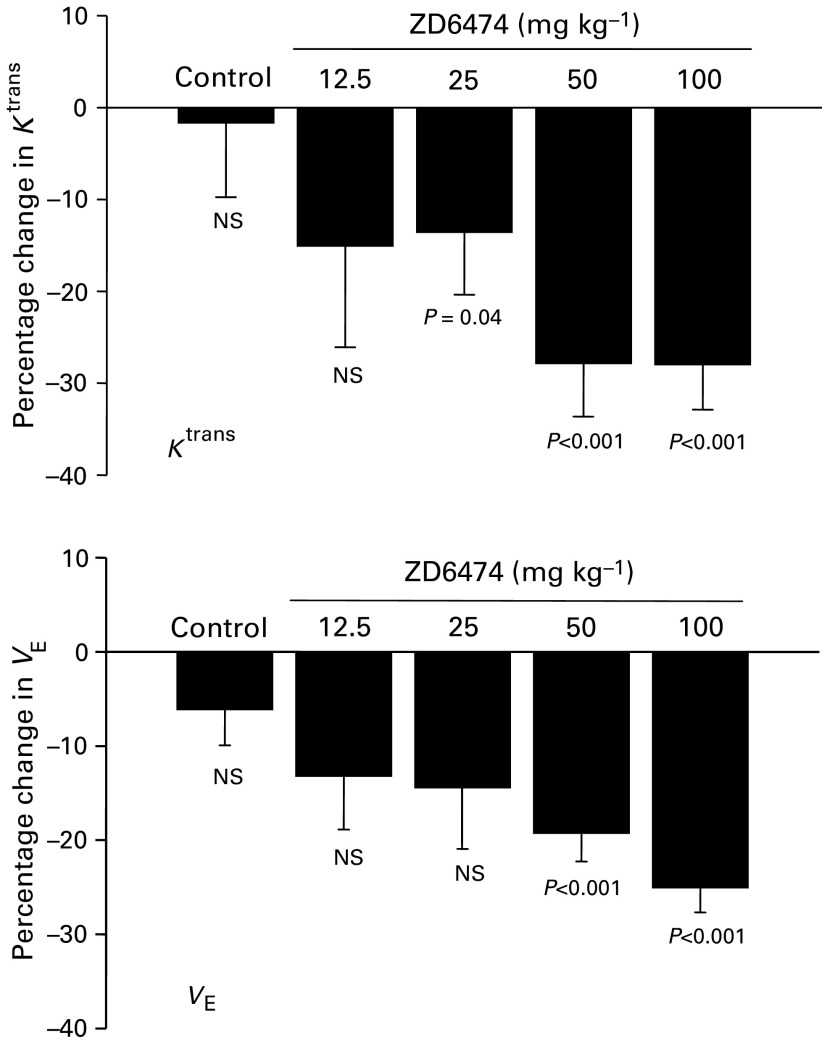
The percentage reduction in *K*^trans^ and *V*_E_ following the treatment of PC-3-xenografted tumours with ZD6474 or vehicle. ZD6474 (25–100 mg kg^−1^) given orally 24 and 2 h prior to imaging. Values are means and s.d. of 6–11 mice. Statistical significance was determined using a paired *t*-test.

).

### Analysis of contrast enhancement using a histogram analysis

**Figure 5:**
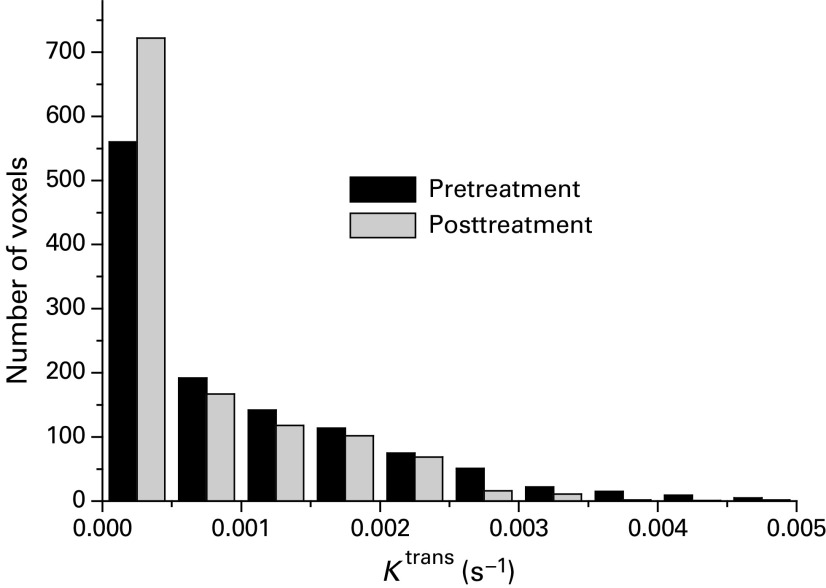
Histogram of *K*^trans^ data for a single tumour pre- and 24 h post-treatment with 50 mg kg^−1^ ZD6474. The total numbers of voxels pre- and post-treatment were 1198 and 1210, respectively. The 20% threshold value for the tumour illustrated was 0.00155 s^−1^.

**Figure 6:**
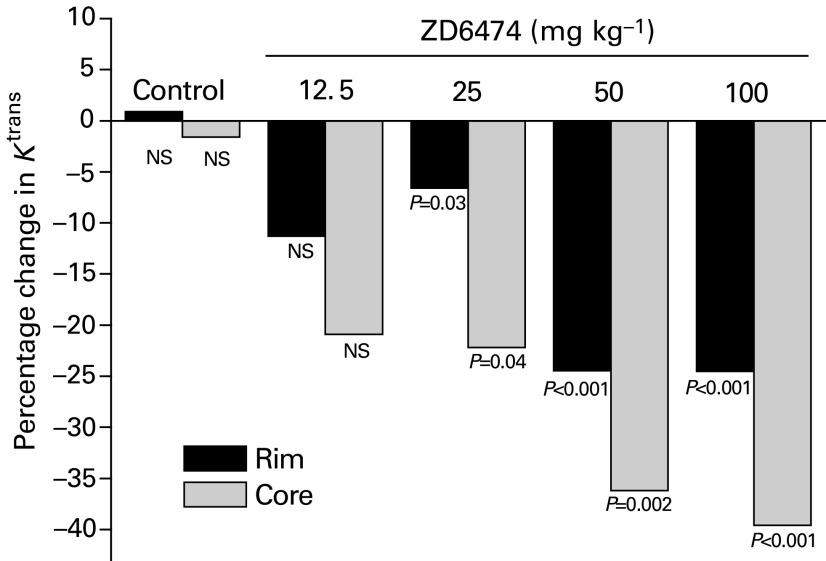
Significant reductions in *K*^trans^ were seen in both the 20% most enhancing (rim) and the least (core) enhancing parts of tumours. Data from a single experiment were analysed using a histogram threshold technique to separate the 20% highest values for *K*^trans^ from the remaining values that corresponded with the tumor rim and core, respectively. Values are means from 6–11 mice, and statistical significance was determined using a paired *t*-test comparing pre- and post-treatment with ZD6474/vehicle.

A histogram analysis was carried out in order to highlight any regional variation in enhancement. Figure 5

shows a histogram of the *K*^trans^ data for a single tumour and illustrates the drug-induced changes seen. Using the histogram analysis approach described in the Materials and Methods, significant reductions in *K*^trans^ were seen in both the 20% most-enhancing (rim) and the remaining least-enhancing (core) parts of tumours (Figure 6

). Larger reductions were seen in the core compared to the rim regions.

### Effect of chronic ZD6474 treatment on PC-3 tumour volume

**Figure 7:**
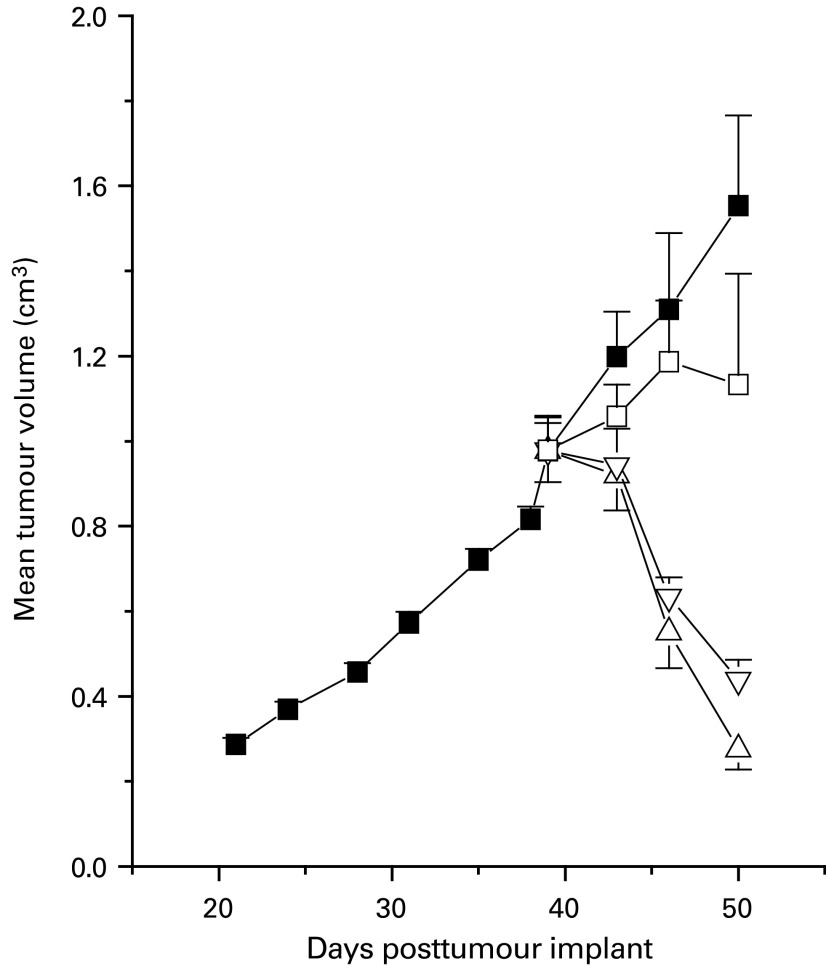
Effect of chronic ZD6474 administration on well-established (1 cm^3^ volume) PC-3 tumours. Oral treatment with vehicle (▪), or ZD6474 (□, 25  kg^−1^ day^−1^; ▿, 50 mg kg^−1^ day^−1^; ▵, 100 kg^−1^ day^−1^) began at day 39.

Chronic once daily oral administration of ZD6474 for 11 days produced dose-related effects on the growth of well-established (∼1 cm^3^ volume) PC-3 tumours (Figure 7

). Marked regression was induced in all tumours with 50 and 100 mg kg^−1^ day^−1^ ZD6474. Although the extent of tumour regression induced by 100 and 50 mg kg^−1^ ZD6474 was similar for the first 7 days of dosing, the response was significantly different following 11 days of treatment (*P*=0.04, one-tailed *t*-test), with mean tumour volumes being 70 and 51% smaller, respectively, than their pretreatment volumes. In contrast, the predominant effect from treatment with 25 mg kg^−1^ ZD6474 was a reduction in the rate of tumour growth, with regressions (of 13 and 40%) only being evident in two out of seven tumours.

## DISCUSSION

Inhibition of VEGF signal transduction is being avidly pursued as a key antiangiogenic strategy, with the potential to constrain or impede the progression of all solid tumour disease. In addition to its mitogenic and motogenic effects on endothelial cells, VEGF can also increase the permeability of vascular endothelium significantly (Senger *et al*, 1990). It may therefore be possible to monitor changes in vascular permeability in response to anti-VEGF therapy, before the consequences of an antiangiogenic effect are apparent. Measures of vascular permeability have been made previously in preclinical tumour models with DCE-MRI methodology and linked to VEGF expression (Bhujwalla *et al*, 2001, Dafni *et al*, 2002; Gossmann *et al*, 2002). Furthermore, clinical DCE-MRI measurements of exchange rates in tumours have been found to correlate with VEGF expression (Knopp *et al*, 1999).

The DCE-MRI study described here used Gd-DTPA to measure the dynamic parameter *K*^trans^ according to the model of Tofts and Kermode (1991). *K*^trans^ reflects the concentration and flow of contrast agent in plasma, the tumour vascularity, vascular surface area, vascular permeability and the size of the extracellular space. This parameter may therefore be appropriate for measuring changes that occur in response to treatment with VEGF-signalling inhibitors. Although low molecular contrast agents have been suggested to have limitations when measuring vascular permeability (Brasch *et al*, 2000), *K*^trans^ was measured reproducibly using Gd-DTPA, with intra-animal variability being comparatively low (CV of 18%). A reproducibility study examining vascular permeability measurements in patients with solid tumors indicates that DCE-MRI assessment using Gd-DTPA may be even more reproducible in man (Jackson *et al*, 2003).

ZD6474 is a VEGF-signalling inhibitor that has demonstrated broad-spectrum activity in established tumour xenograft models, with chronic, once-daily oral administration producing significant growth delays at doses of 25 mg kg^−1^ day^−1^ or less (Wedge *et al*, 2002). In large, well-established, PC-3 tumour xenografts (∼1 cm^3^ volume), it is possible to induce regression with chronic ZD6474 treatment. This phenomenon is believed to result from the inhibition of VEGF-signalling alone (Wedge *et al*, 2002). Since we have found the PC-3 tumour model to be more highly permeable than other tumour xenografts (Checkley *et al*, 2003), it was selected to explore dose-dependent changes in *K*^trans^.

Dynamic contrast-enhanced magnetic resonance imaging enabled an effect on *K*^trans^ to be measured before any inhibition of tumour growth could be detected. Acute dosing of 50 and 100 mg kg^−1^ ZD6474 produced comparable changes in *K*^trans^ (reduction of ∼30%), while chronic treatment induced tumour regressions, with equivalent effects being apparent after 7 days of dosing. However, 100 mg kg^−1^ ZD6474 did eventually have a greater effect on tumour volume, after 11 days of treatment. A smaller but significant effect on *K*^trans^ (reduction of 12.5%) was apparent following acute treatment with 25 mg kg^−1^ ZD6474, but without a significant effect on *V*_E_. The predominant effect of chronically administering this dose of ZD6474 was to inhibit the rate of PC-3 tumour growth. In contrast, acute treatment with 12.5 mg kg^−1^ ZD6474 did not produce a statistically significant effect on *K*^trans^ or *V*_E_. Collectively, these data suggest that in this tumour model, a relationship exists between the dose of ZD6474 administered, the MRI parameters *K*^trans^ and *V*_E_ (measured after acute treatment) and the effect on tumour growth (following chronic dosing).

*V*_E_ is unlikely to be used to monitor changes in vascular permeability, but is reported for interest. Since acute exposure to ZD6474 (50 and 100 mg kg^−1^) would not be expected to reduce the volume of the interstitial space significantly, the reductions in *V*_E_ observed might be a consequence of significantly reduced vascular permeability, which could limit contrast agent availability to the extracellular space. In principle though, *V*_E_ could increase or decrease in response to changes in *K^trans^*, and *V*_E_ is not well determined where *K*^trans^ is low. Indeed, some high values for *V*_E_ were observed in the tumour core where *K*^trans^ was reduced.

This is the first preclinical study to indicate that dose–response information *vs K*^trans^ can be obtained acutely with a VEGF-signalling inhibitor, using Gd-DTPA. Small molecular weight contrast agents have recently been used in two other preclinical studies to examine the effect on *K^trans^* following inhibition of VEGF signalling. In the first of these, an antibody to VEGF was administered to athymic rats bearing small intracranial tumours (Gossmann *et al*, 2002). A very significant reduction in *K*^trans^ was measured, although the effect was assessed following three doses of antibody over the course of a week, and the tumours may have been more highly perfused given their small size and site of growth. In the second study, a mixed KDR and Flt-1 tyrosine kinase inhibitor was assessed at a single dose (50 mg kg^−1^ day^−1^) following chronic administration (21 days) in a murine syngeneic renal tumour model and a reduction in permeability of approximately 50% was reported (Drevs *et al*, 2002a). Interestingly, ZD6474 has also demonstrated a highly significant effect on tumour growth and metastasis in this particular model (Drevs *et al*, 2002b). Collectively, these data highlight a growing interest in the use of clinically applicable contrast agents to measure *K*^trans^ and determine responses following acute treatment with VEGF-signalling inhibitors.

Analysis of the mean of all voxels within a tumour may not be optimal for assessing the effectiveness of anti-VEGF therapy using DCE-MRI, since highly vascular areas could respond differently from regions containing substantial necrosis. The tumour rim is known to be highly enhancing, and the centres of tumours are known to contain variable degrees of necrosis (Marzola *et al*, 2003). However, methods for analysing groups of *K*^trans^ maps are not well developed and generally manually selected ROI are used. Examination of the distribution of data using a histogram analysis, as used in this study, could provide additional information on intratumour heterogeneity, reduce sampling errors and improve understanding of the mechanisms behind tumour responses to treatment. The histogram threshold analysis indicated that, following ZD6474 treatment, significant reductions in *K*^trans^ were evident in both areas of the tumour, but tumour cores were affected to a greater extent. Using manually selected ROI in xenografted human breast tumours, reduced vascular permeability has been seen to a similar extent in both the tumour rim and its centre, following administration of an anti-VEGF antibody (Pham *et al*., 1998). The disparity between this study and that reported here may be attributable to differences in anti-VEGF agent, the method of sampling and analysis, choice of contrast agent, or simply highlight the potential for model-dependent effects.

In summary, this study suggests that DCE-MRI, using a small molecular weight contrast agent, could provide a method for examining permeability changes in response to acute treatment with an inhibitor of VEGF-signalling. The study also indicates that a histogram analysis of tumour *K*^trans^ data can reveal additional spatial information of interest.
